# Facilitating access to supervised smoking facilities: a qualitative study

**DOI:** 10.1186/s12954-025-01217-9

**Published:** 2025-04-18

**Authors:** Courteney Wettemann, Jeffrey Morgan, Tara Taylor, Ardina Boll, Scott Maxwell, Lindsey Richardson, Seonaid Nolan, Jenna van Draanen

**Affiliations:** 1 Department of Child, Family, and Population Health Nursing, University of Washington, Washington, Health Sciences Building, Box 357262, Seattle, Seattle, WA 98195 USA; 2 Research with Expert Advisors on Drug Use, University of Washington, Washington, Health Sciences Building, Seattle, Seattle, WA 98195 USA; 3 BC Centre on Substance Use, 400 - 1045 Howe Street, Vancouver, BC V6Z 2A9 Canada; 4 School of Population and Public Health, University of British Columbia, 2206 East Mall, Vancouver, BC V6T 1Z3 Canada; 5 Overdose Prevention Participatory Research Assistant Program, Overdose Prevention Society, 58 E Hastings St, Vancouver, BC V6A 1N1 Canada; 6 Spencer Creo Foundation, 500-610 Main St, Vancouver, BC V6A 2V3 Canada; 7 Faculty of Arts, Department of Sociology, University of British Columbia, 6303 NW Marine Drive, Vancouver, BC V6T 1Z1 Canada; 8 Faculty of Medicine, Department of Medicine, University of British Columbia, 2775 Laurel Street, Vancouver, BC V5Z 1M9 Canada

**Keywords:** Supervised consumption site, Harm reduction, Implementation, Peer worker, Equity

## Abstract

**Introduction:**

The implementation of supervised smoking facilities (SSFs) as a harm reduction intervention has public health benefits for people who inhale drugs, but there are significant knowledge gaps surrounding the perspectives of SSF visitors and staff on their implementation and accessibility. We conducted this study to learn about their perspectives on barriers and facilitators to accessibility at SSFs.

**Methods:**

The study used a community-based participatory research study design. PWUD and SSF leadership were involved in all phases of the research project as members of the research team. Between June 2021 and April 2022, we conducted 10 qualitative semi-structured interviews with peer workers and stakeholders at an SSF in Vancouver, Canada to examine perspectives on how to facilitate accessibility for visitors. Interviews were analyzed using an abductive analytic approach, themes were defined and organized collaboratively by the research team.

**Results:**

Peer workers and SSF leadership interviewed in the study described aspects of the SSF that contributed to a low-barrier service model and resulted in greater accessibility for visitors, including: (1) non-punitive approaches to interpersonal challenges, (2) anonymity and privacy, (3) peer involvement, and (4) physical environment. Limitations to access were also described and included: (1) age restrictions, (2) geographical location and (3) infrequent, temporary bars for certain behaviors.

**Discussion:**

Findings from this study identified many dimensions of the low-barrier SSF service model and site design that may contribute to greater accessibility for SSF visitors. Findings from this study could be used to inform the scale-up and implementation of SSFs as a harm reduction approach to reducing mortality and other negative outcomes related to the current drug toxicity crisis.

## Introduction

Overdose prevention sites (i.e. “supervised consumption sites”, referred to here as OPSs) are an evidence-based harm reduction strategy where visitors can use drugs under the supervision of staff and volunteers who are trained to identify and respond to overdose events (Kerr et al. 2017; Levengood et al. 2021; Potier et al. 2014; Pauly et al. 2020). OPSs may also offer a suite of important services in conjunction with supervised consumption, including case management, housing resources, wound care (Levengood et al. 2021), referrals to substance use treatment (Potier et al. 2014), and access to basic needs like food, clothing, and hygiene resources. OPSs can function as a community space and are a place where people can use drugs without being scrutinized by the public or criminalized by law enforcement. (Levengood et al. 2021; Potier et al. 2014). OPSs have shown positive social impacts including broadening community support and connectedness and creating opportunities for people who use drugs (PWUD) to be employed as peer workers. (Kerr et al. 2017). Additionally, OPSs have been shown to decrease criminal-legal involvement and interpersonal violence among those that use their services (Collins et al. 2019). OPSs in North America have historically catered only to people who inject drugs. Now, a growing number of OPSs in North America (mostly located in Canada, like the site in this study) are expanding to offer supervised smoking facilities (SSFs) or “inhalation sites” to meet the needs of people who smoke drugs (Kerr et al. 2017). SSFs are often co-located with facilities for supervised injection (Jozaghi & Vancouver Area Network of Drug Users, 2014) and offer spaces where people can smoke drugs under supervision of peer workers, as well as access safer use supplies (e.g., pipes), peer support, and other resources (Kennedy et al. 2020).

Currently, SSFs are struggling to expand and meet rising demand for people who smoke substances. Indeed, as others have noted, smoking has become an increasingly preferred route of drug administration among PWUD (Kerr et al. 2017), for reasons such as ease of administration, harm reduction, ease of access to equipment, decreased stigma, and concerns related to overdose, although there is little evidence that smoking reduces the risk of overdose (Kral et al. 2021; Papamihali et al. 2020; Watson et al. 2013). Research also suggests that smoking substances (over injecting) decreases the likelihood of sustaining soft tissue injuries like abscesses and may reduce the transmission of infectious diseases associated with injecting drugs, like HIV and Hepatitis C. (Kral et al. 2021; Pauly et al. 2020).

There is demand for evidence to inform the scale-up and implementation of SSFs, including awareness of strategies to increase the accessibility of these critical harm reduction services. While there have been studies examining OPS accessibility for people who inject drugs, qualitative research on SSF accessibility for people who smoke drugs remains an important knowledge gap (Potier et al. 2014). This qualitative study examines factors that influence site accessibility from the perspectives of peer workers, staff, and volunteers at a low-barrier SSF in the Downtown Eastside of Vancouver, Canada, an area with a high concentration of services for PWUD.

## Methods

### Community-based participatory research design

This study, based in Vancouver, BC, utilized a community-based participatory research study design with PWUD as community co- researchers. CBPR is a collaborative approach to research that emphasizes the participation and influence of communities affected by issues being studied and aims to meaningfully involve community members in every aspect of research. Key principles of CBPR adopted in this study included power sharing, co-learning and the bi-directional exchange of knowledge, reciprocity, and a commitment to addressing inequities (Beck McGreevy et al. 2023)

This study was part of a broader community-based research collaboration between the University of British Columbia, University of Washington, and the SSF that began in 2017. The research collaboration included a community-based research initiative known as the Overdose Prevention Peer Research Assistant (OPPRA) Project, which had a broad mandate to engage PWUD and peer workers in research about harm reduction with a strong focus on research capacity building and knowledge exchange. The OPPRA Project included senior leadership (TT) and peer workers with the SSF and PWUD (SM), and academic researchers (JM, SN). In 2020, academic researchers and the SSF collaborated on a successful grant to document the lifesaving harm reduction work led by the SSF and advocate for addressing important gaps in harm reduction for people who inhale drugs.

Leadership at the SSF where data collection was performed were involved in identifying project aims and research objectives: examining the current service model, identifying areas of improvement, and creating recommendations for future development and implementation. Community co-researchers who participated with the OPPRA Project were heavily involved in developing the qualitative interview guide, implementation of the study, and the interpretation of research findings. Specifically, OPPRA Project participants engaged in hands-on capacity-building workshops on qualitative research, interview guide development, semi-structured interviewing, and thematic analysis.

Draft research materials, such as the qualitative interview guide, were reviewed together with co-researchers who provided feedback and brainstormed additional questions.

To preserve the integrity of the data and the anonymity of interviewees, interviews were conducted by researchers who did not work at or visit the SSF, as such co-researchers were not involved in data collection. Preliminary qualitative findings were shared back with OPPRA team members, who reflected on findings in relation to their experience as peer workers and offered new areas for further inquiry. OPPRA team members were also involved in the drafting of this report.

### Data collection

Between June 2021 and April 2022, researchers conducted qualitative interviews with ten individuals involved with operating the SSF in various capacities, including as peer workers (many of whom are also site visitors), supervisors, and members of the Board of Directors. Most of the interviewees identified as PWUD, including supervisors and members of the Board of Directors. Interviewees were predetermined collaboratively by the research team and site management and were selected using purposive sampling to maximize diversity in organizational perspectives. Recruitment involved a manager at the site providing information about the study to selected potential participants who were invited to contact researchers if they were interested in participating, clearly communicating privacy and confidentiality safeguards, and ensuring potential participants understood that participation was voluntary and independent of their work with the SSF. Researchers conducted an informed consent process and scheduled interviews with interested participants. Eight semi-structured interviews were held over the phone and two interviews were held in person using a topic guide that was informed by existing research on barriers and facilitators to service access and was pilot tested by community co-researchers. All interviews were audio recorded and transcribed verbatim. Interviews lasted between 35 and 75 minutes. This study was reviewed and approved by the University of British Columbia Behavioral Research Ethics Board.

We asked interviewees about a variety of topics related to their experience working at the SSF including roles and duties; the peer run model of the site; experiences delivering indoor injection and outdoor smoking site services; relationships between the SSF, visitors, and community stakeholders; and the impact of COVID-19 on site operations and harm reduction service provision more generally. To preserve participant anonymity, demographic data was intentionally not collected and is therefore not reported in the results. The sample was drawn from a small pool of peer workers at the same site, so interviewees would have been easily identified with minimal descriptors. Some of the staff interviewed also worked at the OPS co-located with the SSF but were asked to focus on their work at the SSF for the purpose of this study.

### Data analysis

Data analysis began with a transcript review, with each transcript independently reviewed and verified against audio recordings by at least two members of the study team (coders) to ensure data quality. During this review, coders noted impressions and patterns for thematic analysis (Clarke and Braun 2017) using an abductive approach (Timmermans and Tavory 2012) that moved between themes derived from pre-existing literature and emergent themes in the data with a literature review and the research aims as a guide.

During the first round of coding, each reviewer categorized the data using the identified concepts and brainstormed potential codes. Reviewers then met as a group to compare their impressions and create an initial codebook and code descriptions, which were used by each reviewer to separately code the same transcript and ensure mutual understanding of the data. After this second round of coding, reviewers met again and collaborated to finalize the codebook.

The final codebook was used to analyze each of the transcripts, with two reviewers coding each transcript using NVivo 12. Once each transcript was coded twice independently, the group met again to resolve any discrepancies in coding and discuss emerging patterns in the dataset. Using excerpts from the dataset, the group compiled a list of themes and subthemes, comparing them against the data set to ensure coherence. Themes and subthemes were then formally defined and named.

## Results

Interviewees in this study referenced the SSF’s uniquely low-barrier service model throughout the interviews and described how they felt aspects of this model contributed to the accessibility of the site. The association between the low-barrier model and site accessibility in the interviews was strong enough to be synonymous, as illustrated by the interviewee below:

“*Like it’s always super accessible. We’re like the lowest-barrier site. Like I always joke*,* you know*,* so many sites are low barrier. I’d say we’re like no barrier. Like absolutely no barrier.” - Interview 1*.

Broadly, interviewees characterized a low-barrier model as service provision designed to reduce or remove social and practical barriers to access for both visitors and staff. Through qualitative thematic analysis, we identified several dimensions of access that participants identified as contributing to the low-barrier model:

1. Non-punitive approaches to interpersonal challenges.
2. Anonymity and privacy.
3. Peer involvement.
4. Physical environment.

In a concluding theme, we also describe the Limits of Access: barriers that continued to impede access and areas to develop the low-barrier models of care.

### Non-punitive approaches to interpersonal challenges

The SSF’s approach to interpersonal challenges was one of the most frequently cited elements of the low-barrier SSF model and increased accessibility among site visitors. Interviewees discussed the benefits and drawbacks of its flexible approach to resolving conflict among site visitors and between site visitors and staff and emphasized the site ethos of inclusivity of everyone. Peer workers at the site said they work to accommodate people who struggle to meet the expectations of other SSFs because of perceived disruptive behavior.

In the event of serious infringements on the rights of other SSF visitors like theft or violence, consequences are reviewed on a case-by-case basis and can involve conversations between site staff and visitors to determine why the behavior is happening and how to resolve it so all parties can continue using the site. SSF workers generally try to resolve conflict through dialogue or request the people involved leave the site for a determined period; interviewees agreed this approach is usually effective. After the break, the individual is welcomed back, as illustrated by the quote below:

*“It’s very*,* very*,* very hard to get kicked out of [the site]. We never*,* ever say*,* “You’re banned*,*” we just say*,* “You need to take a break.” We use specific language*,* so that there’s not a permanent end to it. We just say*,* “You’re causing some problems around here*,* you need to take a break.” You know*,* absence makes the heart grow fonder. If you disappear for a week*,* we’ll be happy to see you when you come back”* - *Interview 1*.

They stated that most often, conflicts among visitors at the site are minor and can be resolved through conversation. Several interviewees described a careful delineation between behavior that felt disruptive and behavior that posed a real danger to others. One interviewee described an interaction with a visitor who was digging through the garbage onsite: after asking them to stop multiple times, the interviewee stopped trying to manage the behavior, feeling that it was not necessary to punish them for doing something that was ultimately harmless. As one interviewee described:

*“We don’t have many rules. Like there’s only basically one rule*,* and it’s no violence onsite.*” - *Interview 7*.

Interviewees felt this approach contributed to positive relationships among staff and site visitors. Some described a collective effort between SSF peer workers and SSF visitors to work together to resolve conflicts collaboratively. At the time of data collection, none of the interviewees described an instance where a visitor’s behavior had warranted a permanent bar.

Interviewees said the flexibility with respect to interpersonal conflict and disruptive behavior is necessary considering how many visitors face prohibitions from accessing harm reduction services, mental health care, and other resources elsewhere, and recognized the chronic stress that many of their visitors experienced as members of a stigmatized and criminalized community. Interviews revealed that site staff understood the impact of traumatic life events like institutionalization and homelessness, sometimes through their own first-hand experience, and were accepting of behaviors that could be explained as a reaction to stress.

Several interviewees said they resisted barring people from the site out of concern that visitors would not be able to access other harm reduction services. Interviewees were concerned about the potentially fatal consequences of turning people away given the unpredictable and toxic nature of the unregulated drug supply.

“*We’re the only ones that – like we’re the only one that’s basically a no-barrier […] site. Because if we didn’t have this one*,* there would be a lot of people that wouldn’t be able to go into the other ones… without us*,* there’s a lot of people that would be using in the alleys.” -Interview 9*.

### Anonymity and privacy

In line with harm reduction best practices, the SSF eliminated documentation barriers and respected visitors’ anonymity by using pseudonyms or “handles” instead of real names. Interviewees viewed expectations to provide identifying information as intrusive, prohibitive, and unreasonable, as described by the quote below:

*“We never had a – we never did the passport thing*,* because we just thought it was too big of a barrier for our [visitors]*,* you know*,* especially if you need picture ID and stuff*,* right*,* which goes against our whole point of anonymity*,* right?” -*Interview 10.

As such, interviewees reported that the SSF broadened access from the first encounter by not turning anyone away at the door for not supplying personal information. Interviewees felt that welcoming visitors into the site without requiring anything from them lowered barriers and set a precedent establishing that privacy and confidentiality is respected in the space. While the practice of preserving anonymity is not unique among SSFs, it was brought up frequently by interviewees as preferable to other services geared towards PWUD that do not prioritize participant anonymity.

Multiple interviewees discussed their perceptions of how the layout of the SSF influenced visitors’ feelings of privacy and anonymity. While they said some visitors prefer to feel a sense of privacy while they use drugs, the ability for staff and visitors to co-monitor each other is a necessary part of the design of the space, making it possible to respond when a visitor is overdosing.

*“From the time OPS started to*,* say*,* now in those four years*,* people understand a lot more of why we can’t have tarps*,* solid tarps*,* and curtains and this blocking the sides*,* for overdose reasons. And even they learned*,* they learned to just accept that because*,* you know*,* it’s our job to make sure while you’re on this property*,* that you don’t die using these drugs […] That’s why I’m saying*,* like maybe clear plastic tarps or whatever*,* that still give the feeling of privacy*,* but the surrounding*,* but you can still see through it for emergencies.”-Interview 9*.

As the interviewee above describes, maintaining visitors’ privacy and safety simultaneously was seen as a challenge.

One drawback to visitors’ privacy was the location of the SSF in a lot surrounded by high-rise buildings. Interviewees said that visitors were sometimes visible to residents of the apartments above, which was described as occasionally bothersome to both the visitors and residents.

### Peer involvement

The SSF staff include peer workers across the organization, and day-to-day operations and management were primarily run by peer workers. Interviewees said that as peer workers, they could positively impact service provision through compassion informed by personal and lived experience, including insider knowledge on harm reduction and drug supply and an intimate understanding of the factors that impacted day-to-day life for PWUD. They cited several ways that peer workers increased accessibility: making the SSF more comfortable through rapport, resolving conflict more effectively, and using knowledge of barriers to improve service access. The quote below demonstrates this concept:

*“What we’ve heard is that it’s a preference…being run by peer [worker]s. Because when you don’t – and this is like this is no shade or anything to like any of the health professionals*,* but*,* you know*,* when you have unions and stuff involved*,* you can only respond in ways that are etched out by generally people who don’t know or understand the frontlines. And so*,* by having it being fully peer response*,* […] There’s also a lot more compassion and understanding where maybe people are coming from*,* when you’re having a hard day and someone who’s maybe not been where you are tries to tell you something*,* it can be received as like condescending.” -Interview 1*.

This excerpt also describes the benefit of including peer workers at the leadership and direct service level. Several interviewees touched on this, stressing the importance of decision-makers having a thorough understanding of drug user experiences of service and accessibility to inform site policies.

Peer workers at the direct service level were also able to influence service design and delivery to improve accessibility. Many of the peer workers in direct service roles who were interviewed were also visitors to the SSF, giving them a dual perspective of the site’s services. Peer workers in this study described how the low-barrier policy for site visitors extended to the workplace as well, as described by the interviewee in the quote below:

*Sometimes the staff are having a bad day… We have an open-door policy for hiring staff*,* and they might be institutionalized*,* or they might have mental health issues too. So*,* you know*,* something I learned working here is*,* you know*,* sometimes you just need to go for a walk or something*,* for staff members […] it doesn’t mean you did anything wrong […] that is something we really try and focus on too*,* is that you didn’t do anything wrong just for an outburst of anger or frustration or whatever*,* right?” -Interview 4*.

Having a low-barrier workplace was seen by interviewees as a way of creating a staff that shared many elements of identity with site visitors, benefiting visitors through shared understanding and the ability to build rapport. Many of the interviewees who discussed the employment policy had mixed feelings, though, seeing the benefits and drawbacks as two sides of the same coin: for example, one interviewee spoke about how site staff may also face some of the same frustrations and barriers that are common among non-staff site visitors, like previous institutionalization or lack of access to housing, sometimes impacting their work. However, none of the interviewees felt that the hiring policy should exclude peer workers who used the site.

Some interviewees suggested that worker demographics also played a role in accessibility. They described their perception that peer workers who shared aspects of identity with visitors (such as gender identity, racial identity, Indigeneity, etc.) had the positive impact of more easily developing rapport and increasing visitors’ feelings of belonging.

In the interviews this sentiment was mostly strongly associated with gender, particularly among women employed as peer workers at the site who stated their belief that female-identified visitors felt more comfortable around staff with the same gender identity. When asked how the SSF meets the needs of women, an interviewee who identifies as a woman responded:

*Sometimes the women need feminine products*,* right*,* and they’re not going to go up to a man. You know*,* that’s why it’s good to have female workers because of those situations. And then there’s some of the women [visitors]*,* they don’t want to talk to any guys. They just want to talk to women […] I think that […] having women there is very important. -Interview 8*.

Interviewees held similar feelings about racial and gender identities among site staff as they did about peer representation; namely, that holding shared identities with staff at the site increased visitor comfort in accessing services and ease of rapport-building. Seeing representation of a shared identity among people who hold power and provide services at the site functioned as a signal of shared understanding and safety from stigma.

### Physical environment

Interviewees described physical elements of the SSF that impact accessibility. Interviewees noted a preference for a large, airy space, with accessible bathrooms and other amenities to address visitors’ basic needs. The location of the SSF in the Downtown Eastside, an area with a high concentration of services for PWUD, was also frequently cited as a key factor that could dramatically influence how accessible the space was.

The size of the SSF has physical and emotional repercussions as described by the interviewees. Several interviewees described a large space with air flow as more pleasant and less likely to induce claustrophobia and anxiety. Including a “chill area” where visitors have plenty of room where visitors have plenty of room to move around was described as helpful. Room for storage was also frequently cited. Interviewees noted that many of their visitors are unhoused and unwilling to leave their personal belongings behind to use an SSF, so having a space large enough to accommodate tents, large bags, and even pets positively impacted accessibility. The quote below describes in more detail:

*“Some people are also homeless*,* and have a lot of luggage*,* or their possessions. An outside site can accommodate that*,* depending on the size of the site. Some people aren’t used to – or don’t like being inside*,* so having that option of having it outside is great… When we had the larger site*,* we even had a garden growing*,* which a lot of the [visitors] enjoyed. We had*,* you know*,* memorials there for passed – or people who had passed away. We had barbecues set up there*,* because we had a large chill zone sort of area also*,* an area where we can monitor someone if – you know*,* they’re no longer using*,* but we still need to keep an eye on them. So yeah*,* that’s definitely a lot of the great stuff about the outside.”-Interview 3*.

The quote above also illustrates the SSF’s efforts to create a pleasant atmosphere at the site (e.g., garden, barbeques) and gives an example of a benefit of having the SSF outdoors. An outdoor site was effective at promoting air flow and reducing exposure to unwanted fumes from smoked drugs, a common concern voiced among interviewees as SSF visitors bring different substances with varying effects. Several interviewees also felt that an outdoor space was easier to expand and could accommodate more people as demand increases. Interviewees also felt that a large space is easier to navigate for people with limited mobility and for those who use aids like wheelchairs, as well as keeping space open to quickly respond to overdose and other emergencies.

However, there was some disagreement among interviewees over whether an outdoor site was a desirable SSF design characteristic. Some interviewees felt that an outdoor site reinforced stigma against people who smoke drugs and believed it indicated a lack of prioritization of resources for people who smoke rather than inject- making them feel like an afterthought. Interviewees also described the miserable conditions at the outdoor site during inclement weather, especially during the winter. The outdoor areas become uncomfortably cold, wet, and windy at certain times of year, negatively impacting both staff and visitor experiences at the site. While the interviewees suggested that uncomfortable conditions at the outdoor site could be a deterrent for some visitors, they stated that prohibitive costs and logistical barriers prevented serious consideration of building an indoor SSF. These barriers did not apply to the indoor injection facilities as they were specific to the management of air quality in an indoor smoking space.

Many interviewees felt that the existing layout for the SSF, which had separate, designated spaces for smoking and injecting, better accommodated differences between drugs that would be used in each space, as the quote below illustrates:

*“In the injection room*,* basically all they’re doing is injecting it and it doesn’t matter if they’re injecting fentanyl or down…because they’re shooting it in their arm*,* so it’s not bothering anybody else. But the smoke would bother other people.” -Interview 6*.

Having an area for injecting separate from the smoking area was referenced by the interviewees as a strength as it allowed visitors to inject drugs while avoiding exposure to fumes. Interviewees also felt that for visitors who both smoke and inject drugs, having separate spaces allowed them to choose their preferred environment. For example, interviewees said the SSF had more space, fresh air, and a social atmosphere, while the injection site was described as quieter and protected from inclement weather. An interviewee quoted below expands on this:

*“A lot of people tend to inject…their heroin*,* so we see a lot of kind of more quiet behaviour in the injection room […] we see a lot more of like kind of the crystal meth users at the inhalation site. And sometimes the behaviour around that is a little bit more*,* you know*,* enthusiastic.” -Interview 2*.

Interviewees noted the lack of supervised smoking facilities in the area compared to supervised injection facilities and felt that the SSF needed to expand to meet demand as the popularity of smoking increases.

In addition to having space for smoking and providing harm reduction services, the SSF also had bathroom facilities for visitors, which interviewees felt positively impacted visitor comfort and encouraged people to stay as long as they needed. Interviewees noted the lack of sanitation resources in the community despite the SSF being in a busy neighborhood, partially due to discrimination from local businesses against people who are unhoused or using drugs. As such, the SSF fulfilled an important community need beyond its purpose to provide supervised drug use services.

Location was also a principal element of accessibility. The SSF in this study was located in Vancouver’s downtown core, close to other services for PWUD and near a large homeless encampment at the time of data collection. Interviewees felt that most visitors would not walk far to use an SSF, and some would not be able to make it to the site if it were even a couple of blocks further.

### Limits of access

While interviewees felt that the SSF was low-barrier and highly accessible for the majority of site visitors, interviewees identified several barriers to service access that still existed.

One important barrier identified by interviewees was age: the SSF did not allow visitors under the age of 18 into the site. Several interviewees described moral distress about complying with age restrictions, and multiple interviewees expressed concern about the consequences of turning teenagers away from a crucial resource during a prolonged drug toxicity crisis. Interviewees expressed discomfort with allowing children into the site but felt it was not helpful to deny access. Interviewees noted that anyone under the age of 18 has no options for supervised use in the area, and that there is a gap in services for youth who use drugs in general.

While the SSF was considered a very low-barrier site by the peer workers interviewed in this study, the site did temporarily “bar” people occasionally in cases of repeated threats towards visitors or staff. Interviewees described mixed feelings around the use of even temporary bars, noting that if people were barred from the SSF, they were likely unable to access other sites as well. These cases were infrequent enough that interviewees did not see this as a significant barrier. Discussion centered around balancing accessibility with the need to preserve a safe space for all visitors and staff. On the other hand, interviewees acknowledged that a low-barrier approach could also be a deterrent for visitors if low-barrier policies were perceived to expose visitors to violence, theft, or other harm.

Finally, interviewees felt that the SSF’s location in the downtown core close to other services like supportive housing.

### Summary

Overall, in our study, interviewees articulated a shared understanding of the low-barrier model that they felt made the site inclusive, especially for people who may experience barriers accessing other SSFs and harm reduction services. Specifically, this included site policies that eliminated social and practical barriers to entry and took non-punitive approaches to conflict resolution that did not result in service restriction. Having peer workers staff the site was a commonly and strongly referenced element of the SSF that increased accessibility for visitors and contributed to a low-barrier service model. Physical considerations that facilitated access and comfort included large open spaces with bathrooms for visitors to use, and the geographic location of the site close to other services. Still, despite sentiments about the site having few barriers and being generally highly accessible, some limits to access were noted for youth, people who do not live in or normally visit the Downtown Eastside, and those needing services at night.

## Discussion

Our study conducted interviews with peer workers at a low-barrier SSF in Vancouver, BC to learn about the factors that influenced accessibility and among PWUD willingness to access the site. Because the SSF uses a peer-led model, many of the interviewees were also visitors and provided nuanced, multi-faceted perspectives on barriers and facilitators to accessibility at SSFs from their multiple viewpoints: a strength of the study and a departure from normative data collection about service use and delivery.

Findings in this study suggest that flexible, low-barrier services and peer representation at SSFs may influence PWUD willingness to access those services. As many of the site visitors are subject to criminalization and surveillance outside the site because of their drug use, the function of the SSF as a “safe space” where visitors can use drugs without stigma or fear contributed to visitors’ desire and willingness to access the site. PWUD encounter various forms of stigma in service provision settings, with evidence showing that stigmatizing experiences decrease PWUD willingness to seek services (Biancarelli et al. 2019). The service model at this SSF is intended to ameliorate the impact of this stigma.

One of the elements that contributes to this “safe space” was the peer-led model, which emerged as a primary factor in accessibility as described by the interviewees, both in terms of increased comfort for the visitors and as a way to inform service provision with the expertise of lived experience, including reducing barriers that prevent PWUD from accessing services. Previous research has documented the benefits of peer-led models at harm reduction sites and OPSs in the context of injection drug use (Bardwell et al. 2018; Foreman-Mackey et al. 2019; Kennedy et al. 2020; Urbanik and Greene 2021), and our study contributes qualitative data that demonstrates those benefits for accessibility at SSFs, which have many of the same characteristics as OPSs but where services are expanded to meet the needs of people who smoke drugs. As the drug landscape rapidly changes, including which substances are ingested (Kral et al. 2021) and which routes of administration are dominant (Kral et al. 2021; Parent et al. 2021), peer workers are aware of changes as they happen and can adapt services to community needs with more flexibility than other service models (Urbanik and Greene 2021). Interviewees also felt that peer workers showed more interpersonal flexibility with visitors than non-peers, viewing them with more compassion and building rapport more easily, which is consistent with qualitative studies of OPS visitors (Foreman-Mackey et al. 2019; Kennedy et al. 2020; Urbanik and Greene 2021). Interviewees recognized that SSF visitors face disproportionate barriers to resources and systemic marginalization and work to meet visitors “where they’re at” (Woolhouse et al. 2011).

Prior research has been done to examine user preferences for the physical layout of SSFs located within OPSs. (Bourque et al. 2019). Findings from studies on visitor preferences found a preference for separate areas for smoking and injecting. One qualitative study found that some visitors who smoke drugs prefer to avoid seeing people inject because it makes them uncomfortable and were concerned about conflict between visitors experiencing different types of highs (Watson et al. 2013). While our interviewees felt that visitors preferred separate areas for injecting and inhaling, reasons were more related to avoiding unwanted exposure and allowing for different “atmospheres” or social dynamics to emerge in each space. Preference for a spacious, communal smoking area where visitors can socialize, move around, and keep their belongings with them was well-represented in qualitative interviews.

The separation of smoking and injecting space also contributes to accessibility by allowing more capacity for visitors. Interviewees felt the outdoor smoking space could potentially be expanded as it is not confined by the physical limits of a building. Other studies show that capacity impacts accessibility because wait times can deter people from using an OPS. (Kennedy et al. 2020; Papamihali et al. 2020).

It is difficult to speculate about how much of an impact the co-location of the OPS with the SSF had on our results. It is possible that the co-location of the site had a positive effect on accessibility, as some participants may prefer to consume drugs in more than one way (smoking and injecting), however more research is needed to confirm this.

### Limitations

The study had a modest sample size due to the limited number of staff at the SSF. Peer workers who participated in the study were often visitors to the site too, but the perspectives of non-staff visitors are not included. Some factors that have been found to influence SSF utilization in previous quantitative research were notably not mentioned by interviewees in this study, including wait times (Kryszajtys et al. 2022), time limits for using at the site (Kryszajtys et al. 2022), limited hours (Kryszajtys et al. 2022), and preferences for things like privacy and women-only services (Foreman-Mackey et al. 2019; Kryszajtys et al. 2022), which may be unrepresented in the data because of the limited sample size. This study also does not examine the experiences of people unable to access the SSF.

The geographical location and social context of the SSF in this study is unique. Canada, and Vancouver, B.C. in particular, has a progressive approach to addressing drug-related harms relative to the United States (Linden et al. 2013; Nadelmann and LaSalle 2017). The Downtown Eastside area in Vancouver is known for its high density of people experiencing homelessness, PWUD, and those with systemically marginalized identities (Linden et al. 2013). It is also a hub for services designed to meet the needs of PWUD and home to a strong culture of community organizing and peer-led interventions by PWUD (Jozaghi et al. 2014). As a result, service users in the Downtown Eastside have been frequent subjects of research on harm reduction interventions in the past decades, with research participants reporting mixed feelings about their experiences as research participants (Damon et al. 2017; Linden et al. 2013). It is possible that the historical saturation of research in the area may have positively or negatively contributed to participant willingness to engage in this study.

## Conclusions

Despite smoking being a predominant mode of administration that is increasingly linked to fatal and non-fatal overdose, the implementation and scale-up of SSFs in North America has been slow. Wider implementation of SSFs, including couching SSFs within other services, may help to accommodate the range of needs and preferences among people who use drugs (Cortina et al. 2018). Research demonstrates a growing need for SSF implementation and emphasizes their importance as a hub for PWUD to access care and support. (McNeil et al. 2015; Pijl et al. 2023; Tapper et al. 2023). To adequately meet this need, SSFs need sufficient funding for services and staff. Funding for space expansion is necessary to meet increasing demand for services and should account for visitor preferences for inside vs. outside spaces as they may change depending on the site location and season (Pijl et al. 2023). As this study and other studies have shown (Foreman-Mackey et al. 2019), client comfort is key; our study suggests it is crucial to invest in SSFs that are welcoming, inclusive, and non-punitive.

## Future directions for research

Future research could focus on the experiences of non-staff visitors to SSFs, and PWUD who are unwilling or unable to use SSFs. Even very low-barrier SSFs like the site in this study are not accessible or acceptable to some PWUD, including youth who use drugs and parents with young children where childcare is inaccessible. Visitors to the Downtown Eastside may be more willing to access the SSF in this study because of the density of other harm reduction services in the area and the Downtown Eastside’s prominence as a hub of mutual aid and peer organizing. As SSFs continue to be implemented in North America, more research will need to be done to determine their acceptability among PWUD in different social and geographical contexts.

## Data Availability

The datasets used and/or analyzed during the current study are available from the corresponding author on reasonable request.

## Abbreviations

OPS: Overdose prevention site
PWUD: People who use drugs
SCF: Supervised consumption facility
SSF: Supervised smoking facility
